# The effect of serum vitamin D normalization in preventing recurrences of benign paroxysmal positional vertigo: A case-control study

**Published:** 2016

**Authors:** Mahboobeh Sheikhzadeh, Yones Lotfi, Abdollah Mousavi, Behzad Heidari, Enyatollah Bakhshi

**Affiliations:** 1 1.Department of Audiology, University of Social Welfare and Rehabilitation Sciences, Tehran, Iran.; 2 2.Iran University of Medical Sciences, Tehran, Iran.; 3 Mobility Impairment Research Center, Babol University of Medical Sciences, Babol, Iran.; 4 Department of Statistics, University of Social Welfare and Rehabilitation Sciences, Tehran, Iran.

**Keywords:** BPPV, 25-hydroxy vitamin D, Epley Therapy, Recurrent vertigo

## Abstract

**Background:**

Benign paroxysmal positional vertigo (BPPV) is a condition with recurrent attacks in a significant proportion of patients. The present case- control study was conducted to assess the influence of serum vitamin D normalization on recurrent attacks of vitamin D deficient patients.

**Methods:**

Diagnosis of BPPV was made based on history and clinical examination and exclusion of other conditions. Serum 25-hydroxy vitamin D (25-OHD) was measured using ELISA method and a levels of < 20 ng/ml was considered a deficiency of vitamin D. Inclusion criteria were as follows: history of recurrent attacks and serum 25-OHD<20.ng/ml. While the patients with history of trauma, surgery and chronic systemic diseases were excluded. The patients were classified into two groups: treatment and control, intermittently. Both groups received Epley rehabilitation therapy one session per week for 4 weeks but the treatment group received an additional supplement of 50.000 IU of vitamin D (cholecalciferol) weekly for two months to achieve serum 25-OHD ≥ 30 ng/ml and the study patients were followed-up for 6 months.

**Results:**

Twenty-seven patients were allocated to each group. At baseline, serum 25-OHD was similar (10.7±2.3 vs 11.41±1.9, P=0.23). At month 2, serum 25-OHD in the treatment group increased significantly to ≥ 30 ng/ ml, whereas serum 25-OHD in the control group remained unchanged (34.2±3.3 vs 10.6 10.6±2.2 ng/ml, P=0.001). During the follow-up period, attacks of BPPV in the treatment group decreased significantly compared with the control group (14.8% vs 96.3% OR= 0.18, P=0.001).

**Conclusion:**

The findings of this study indicate that the normalization of serum vitamin D significantly reduces BPPV recurrences.

Vertigo, the chief complaint of vestibular involvement in association with other manifestations such as dizziness, disequilibrium, blurred vision is a cause of morbidity and psychological symptoms (1-3). Any of these symptoms may occur in about 70% of patients with benign paroxysmal positional vertigo (BPPV) and in cases with recurrent symptoms, quality of life will be affected significantly (4). In one study, over an average period of 47 months, the recurrence rate was 27% and half of the attacks occured in the first 6 months (5). BPPV is the most common vestibular disease involving 2.4 % of population over a lifetime. It is more prevalent in women especially in the sixth decades of age. Most cases of BPPV are idiopathic (6) with recurrent attacks in about 15% of patients.

In a follow-up study, 50% of patients with BPPV had recurrent attacks over 10 years after disease onset which was more common in women than in men and often occured during the first year after diagnosis (7). 

Diagnosis of BPPV is usually based on history and clinical examination and the mechanism of vertigo has been attributed to calcium debris within the posterior semicircular canal known as canalithiasis. Currently various types of vestibular rehabilitation therapy are used for treatment of BPPV. 

A review of 11 clinical trials compared the effectiveness of Epley maneuver with placebo or no treatment, the maneuver was safe and associated with recurrence rate of 36% (8). In a prospective study, treatment with repositioning maneuver was effective in 88% of patients over 1 year follow up with 7.5% relapses (4). 

In another prospective study of canalith repositioning procedure, 91.3% recovered after 1 or 2 treatment sessions. Over the follow-up periods of 26 and 40 months, recurrences occurred in 26.3% and 50% respectively (9). Similarly, 90% of patients with BPVV responded to physical maneuver with a recurrence rate of 12% over 1.5 -year follow-up period (10).

 Medical therapy is usually indicated for acute episodes of vertigo to suppress vestibular function and alleviate symptoms but in mild episodes of BPPV, medical treatment is not useful (2). These observations indicate that BPPV is a recurrent condition and prevention of recurrence rates requires identification and correction of responsible risk factors. 

A few studies have shown a correlation between serum vit D and recurrences of BPPV (11-13). It was shown that vit D deficiency and insufficiency are indepently associated with BPPV and increase the risk of BPPV attacks (12). On the other hand, the patients with recurrent attacks of vertigo have lower serum vit D than those without recurrences (13). 

Vit D deficiency is highly prevalent in the general population and is associated with the progression of many clinical conditions (14-18). In vit D deficient patients, correction of deficiency resulted in significant improvement on osteoarthritis knee pain, pulmonary function, and muscle strength (19). These observations suggest raising serum vitamin D level to normal condition. In one study, the recurrences of BPPV were improved after correction of vit D deficiency (11). 

Regarding the association of BPPV and vit D deficiency, normalization of vit D deficiency in patients with BPPV deserves further consideration.

Data in this context are scarce and the present case- control study was conducted to assess the influence of serum vit D normalization on recurrent attacks of BPPV in vit D deficient patients. 

## Methods

The study population was selected consecutively according to inclusion criteria among vit D deficient BPPV patients presented to Ayatollah Rouhani Hospital ENT clinics over 9 months period from April 2014 to December 2014. Diagnosis of BPPV was made based on history and clinical examination and exclusion of other conditions. Vit D deficiency was confirmed by serum 25-hydroxyvitamin D (25-OHD) measurement using ELISA method. A level less than 20 ng/ml was considered a deficiency (18). 

All patients had history of at least two or more attacks of BPPV over 6 months prior to inclusion. Exclusion criteria were as follows: history of head and ear trauma, surgery or infectious diseases of the ear, maxillary sinuses and neck areas, chronic hematologic, renal, gastrointestinal, cardiovascular diseases, taking calcium supplements, vit D and drugs that affect vit D metabolism. 

 Data were collected through an interview, clinical examination, and laboratory tests. All patients received Epley rehabilitation therapy one time per week for 4 weeks. The study patients were intermittently allocated to treatment group (Epley therapy + supplemental vitamin D) or control group (Epley therapy alone). 

Serum vit D in all patients and controls was measured at baseline, month 1, month 2, and month 6. Treatment group received 50.000 IU cholecalciferol weekly for two months to raise serum 25-OHD up to 30 ng/ml or higher whereas; the control group received only rehabilitation therapy. 

The number of BPPV attacks in each group was recorded over six months follow-up period. In statistical analysis, the two groups were compared according to presence or absence of attacks of BPPV as well as the number of attacks. Chi square test with calculation of odds ratio (OR) was used for comparison of qualitative variables and student t-test was used for the comparison of quantitative variables. SPSS software was used for statistical analysis.

## Results

A total of 54 vit D-deficient patients were recruited into this study. Characteristics of the patients and controls are presented in table 1. At baseline, the two groups were similar with regard to serum 25-OHD. After treatment with cholecalciferol serum 25-OHD was raised to normal level (> 30 ng/ml) in the treatment group with mean value of 34.2 ± 3.3 ng/ml at month 2 whereas, in the control group, the serum 25-OHD remained at deficient level of 10.6 ± 2.2 ng/ml (P=0.001) (table 1).

During the follow-up period attacks of BPPV, the treatment group was significantly lower than the control group (14.8% vs 96.3% OR= 0.18, P=0.001), indicating that supplemental vit D reduced the risk of recurrence by 82%. 

There was also no significant difference in recurrence rate between male and female patients (P=0.37)

**Table 1:** Characteristics of 25-hydroxy vitamin D (25-OHD) deficient patients^¥^ with benign paroxysmal positional vertigo treated with Epley rehabilitation manouevre with (treatment group) and without supplemental vitamin D (control group

**Variables** **Mean±SD**	**Control** **n=27**	**Treatment** **n=27**	**P-value**
Age, years	48.2±4.8	47.8±5.7	
Sex, females No (%)	14(51.9)	15.(55.6)	0.86
Serum 25-OHD status, ng/ml			
Baseline	10.7±2.3	11.41±1.9	0.23
Month 1	10.6±2.2	19.37±3.3	0.001
Month 2	10.6±2.2	34.2±3.3	0.001
Month 6	11.1±2.3	35.5±2.9	0.001

¥ . Serum 25-hydroxy vitamin D < 20 ng/ml

## Discussion

The results of this study indicated that normalization of vit D deficiency in patients with BPPV significantly reduces recurrent attacks. The population of this study recruited among patients who had recurrent BPPV and The occurrence of current attacks were confirmed prior to inclusion. 

Data in this context are scarce and a few studies have addressed this issue. In a study by Talaat et al. raising serum 25-OHD to greater than 10 ng/ml resulted in substantial reduction of relapse rate of BPPV attacks as compared with <10 ng/ml (20). Buki et al. in a study of patients with frequent recurrence of BPPV found lower level of 25-OHD as compared with the general population and supplementation with vit D prevented the recurrences of vertigo attacks (11). Vit D deficiency is highly prevalent in the general population as well as patients presenting to medical clinics (18, 21, 22). In one study of patients presented to E.N.T clinic, only 3 out of 86 patients who had presented with more throat or upper respiratory infections had sufficient serum vitamin D, while the rest of patients had deficient or insufficient serum vit D levels (22).

 Patients with BPPV have lower bone mineral density than controls (13). Biochemical markers of bone turnover correlate with BPPV (23). In a review of 101 cases, BPPV was more prevalent in women at postmenopausal age (6).Low bone mass and bone fracture are risk factors for BPPV.

 These are also prevalent in postmenopausal women due to reproductive and hormonal changes (24-26). Patients with BPPV particularly older than 65 years are at greater risk of fracture (27). A systematic review of seven studies demonstrated a positive correlation between low bone mass and BPPV especially in older women (28).

Beneficial effect of raising serum vit D in this study is of particular importance because vit D deficiency contributes to the development or progression of osteoporosis and fractures as well as occurrence of several common clinical conditions which may coexist with BPPV (14, 15, 17, 18, 29, 30). Thus, the findings of this study highlight serum 25-OHD measurement in patients with BPPV particularly in patients who do not respond to rehabilitation therapy. Nevertheless, interpretation of these findings should be considered with caution, because the present study has several limitations. 

 Data were obtained by self-report and are particularly subject to bias. Information regarding several clinical conditions such as hypertension, hyperlipidemia and diabetes has not been collected. Either of these conditions may directly or indirectly present with manifestations simulating dizziness or feeling of vertigo and confound the results. Nonetheless, the confounding effects are expected to be minimal since these factors are distributed similarly across the two comparison groups. 

The strength of this study is dependent on the design of this study in which the treatment and control groups were followed longitudinally over six months, although the duration of follow-up was not long enough to generalize the results. Additionally, in this study, the role of seasonal variations on serum 25-OHD over the study period is expected to be minimal because of lack of seasonal vit D variations in the geographic region of this study (31).

In conclusion, this study showed that in patients with recurrent BPPV who are under rehabilitation therapy, raising serum 25-OHD to normal value reduces recurrent rate of BPPV significantly. These findings highlight serum 25-OHD measurement in BPPV. Further studies with larger sample size with prolonged follow-up period is required to support these findings.

## Notes

**Funding:** This paper has been derived from the Audiology Ph.D thesis of Mahboobeh Sheikhzadeh (No: 892503001 ).

## Citation:

Sheikhzadeh M, Lotfi Y, Mousavi A, et al. The effect of serum vitamin D normalization in preventing recurrences of benign paroxysmal positional vertigo: A case-control study. Caspian J Intern Med 2016; 7(3): 173-177.
